# Climate-Induced Changes in Grapevine Yield and Must Sugar Content in Franconia (Germany) between 1805 and 2010

**DOI:** 10.1371/journal.pone.0069015

**Published:** 2013-07-23

**Authors:** Anna Bock, Tim H. Sparks, Nicole Estrella, Annette Menzel

**Affiliations:** 1 Chair of Ecoclimatology, Technische Universität München, Freising, Germany; 2 Institute for Advanced Study, Technische Universität München, Garching, Germany; 3 Institute of Zoology, Poznań University of Life Sciences, Poznań, Poland; 4 Department of Zoology, University of Cambridge, Cambridge, United Kingdom; 5 Faculty of Engineering and Computing, Coventry University, Coventry, United Kingdom; New York State Museum, United States of America

## Abstract

When attempting to estimate the impacts of future climate change it is important to reflect on information gathered during the past. Understanding historical trends may also aid in the assessment of likely future agricultural and horticultural changes. The timing of agricultural activities, such as grape harvest dates, is known to be influenced by climate and weather. However, fewer studies have been carried out on grapevine yield and quality. In this paper an analysis is undertaken of long-term data from the period 1805–2010 on grapevine yield (hl/ha) and must sugar content (°Oe) and their relation to temperature. Monthly mean temperatures were obtained for the same time period. Multiple regression was used to relate the viticulture variables to temperature, and long-term trends were calculated. Overall, the observed trends over time are compatible with results from other long term studies. The findings confirm a relationship between yield, must sugar content and temperature data; increased temperatures were associated with higher yields and higher must sugar content. However, the potential increase in yield is currently limited by legislation, while must sugar content is likely to further increase with rising temperatures.

## Introduction

Climate is one of the key factors influencing grapevine yield and quality [1]–[4]. The timing of grape harvesting has been analysed in numerous publications [5]–[12] but studies using grapevine yield [13]–[16] or wine quality [2], [17]–[19] have been studied to a lesser extent. One reason is that long-term datasets on yield and must sugar content are often difficult to obtain [20].

Climate and weather are the main drivers of grape growth and ripening. In particular, temperature of the whole vegetation period influences harvest date [21] and, therefore, yield and composition. Increasing CO_2_ concentrations may also result in a greater accumulation of fruit and consequently yield [14]. Furthermore, anthropogenic factors affect yield and quality. These include management (pruning, choice of cultivars, soils, fertilisers etc.) as well as the economic, social and political background of the period of study [21], [22]. Studies have emphasised [10], [22] the importance of historical information in building the most robust model of climate reconstruction.

**Figure 1:**
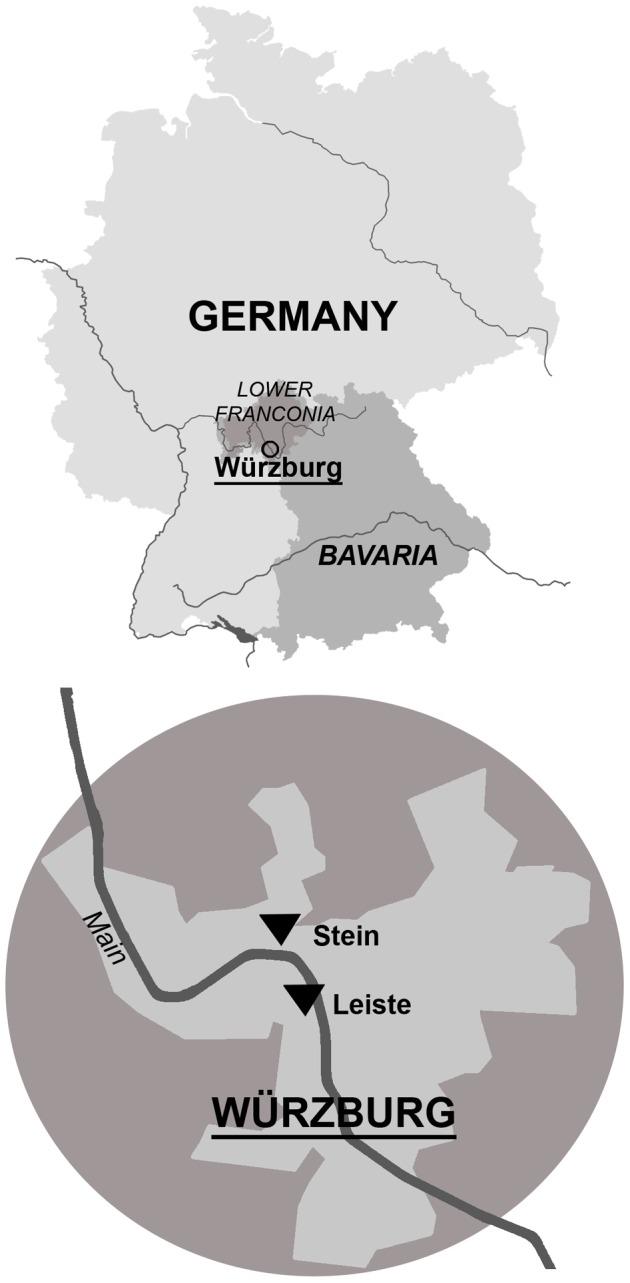
The location of the “Stein” (upper black triangle) and “Leiste” (lower black triangle) vineyard areas used in this study.

Lower Franconia is a long-established (since the 8th century) wine-growing region in Germany (Figure 1) at the northern boundary of grapevine cultivation in Europe. One of the oldest wineries in Germany is the Bavarian State Winery in Würzburg (Staatlicher Hofkeller Würzburg, hereafter Hofkeller), which dates back to the year 1128. Studies using long-term yield and must sugar content of grapes have only rarely been undertaken. Thus, this study aims to add to our knowledge of climate and viticulture relationships by using one of the longest data sets available (1805–2010). The data were obtained from the Hofkeller and are derived from reference vineyards. Records from the nearby Juliusspital winery in Würzburg were used to supplement the Hofkeller data. All records derive from the “Stein” and “Leiste” vineyard areas in Würzburg (Figure 1) and were made by the wineries themselves.

This work aims to evaluate how yield and must sugar content have changed over the recording period. Furthermore, we analyse the effect of temperature on grapevine yield and must sugar content. Therefore we distinguish between the impact of anthropogenic and meteorological factors on yield and composition and estimate the effect of increased temperatures on yield and must sugar content. Thus, the results allow us to assess possible future impacts on the local wine industry.

## Data and Methods

### Grapevine Data

This study analyses long-term time series of grapevine yield and must sugar content in the wine-growing region of Würzburg (49°48′N, 9°56′E) in Lower Franconia, Germany covering the years 1805–2010 (Figure 1). Yield per hectare (hl/ha) and must sugar content in Degree Oechsle (°Oe) at harvest were obtained from the Hofkeller and Juliusspital wineries. Degree Oechsle measures the relative sweetness of the must (grape juice) and shows how much more 1 litre of must weighs compared to 1 litre of water [23]. The data originate from different archive sources but are based on the same vineyard areas (“Stein” and “Leiste”). Grapevine yield data exist for the years 1805–1952 and 1962–2010. The dataset is subdivided into three different, partly overlapping periods (Table 1): 1805–1905 (Hofkeller) +1874–1924 (Juliusspital); 1915–1952 (Hofkeller); and 1962–2010 (Hofkeller). Must sugar contents only exist for Hofkeller, and only for the years 1864–1905 and 1962–2010. Neither yield nor must sugar content data are available for the period 1953–1961. Furthermore, there are a few isolated missing years due to lost or misplaced records.

**Table 1:** Data sources for yield (hl/l) and must sugar content (°Oe) records, identifying the wineries, areas and time periods.

	Variable	Period	Winery	Area	Source
**Period 1**	Yield	1805–1905	Hofkeller	Stein & Leiste	*Eifler (1908)*
	Yield	1874–1924	Juliusspital	Incl. Stein & Leiste	*Weigand (1925)*
**Period 2**	Yield	1915–1952	Hofkeller	Leiste	*Bayerisches Staatsministerium für Ernährung (1977)*
**Period 3**	Yield	1962–2010	Hofkeller	Stein & Leiste	*Annual vintage reports (1962–2010)*
**Period 1S**	Must sugar content	1864–1905	Hofkeller	Stein & Leiste	*Eifler (1908)*
**Period 3S**	Must sugar content	1962–2010	Hofkeller	Stein & Leiste	*Annual vintage reports (1962–2010)*

For period 1, yield (1805–1905) and must sugar content (1864–1905) data for Hofkeller were published by Eifler [24]. Vintage tables for Juliusspital [25] were used to extend the yield time series to 1914. Eifler [24] provides detailed information on yields (hl/ha) for the Würzburg sub-district for almost every year. The Würzburg sub-district includes the famous “Stein” and “Leiste” vineyard areas, which are approximately 2 km apart (Figure 1). These areas consist of several vineyards, which are located on south to south east facing slopes adjacent to the River Main overlooking the city of Würzburg at an elevation of approximately 220–240 m. Eifler [24] also reported, the maximum and minimum must sugar content in°Oe from 1864–1905. During this period, Riesling and Silvaner were the dominant cultivars grown on the “best sites” of the Hofkeller. The original source material no longer exists since it was destroyed during World War II. Weigand [25] lists annual yields per hectare (must in hl/ha) of the Juliusspital winery for 1874–1924. The original Juliusspital source material could not be located and was probably also destroyed during World War II. No data on must sugar content from Juliusspital were available.

Data for period 2 were obtained from a vintage record covering 1915–1952, printed in the “Bavarian agricultural yearbook” [26]. Again, the original source material could not be traced. The record contains annual yield data and acreages of the “Leiste” area. No data on must sugar content were available for this period.

Data for period 3 originate from the annual vintage records (1962–2010) of the Hofkeller that include yield and must sugar content in °Oe for each vineyard separately. To make comparisons with earlier periods, only data from the “Stein” and “Leiste” areas were extracted. For compatibility with period 1, minimum and maximum must sugar content data from Silvaner and Riesling cultivars only were used. Since the records do not contain information on areas cultivated, acreages were estimated by interpolating area data from Eifler [24], Bayerisches Staatsministerium für Ernährung [26], Klopsch [27] and information given verbally by the Hofkeller. For all three periods, a small number of apparently incorrect data (outliers) were checked for plausibility (correlation with neighbouring areas), obvious errors (e.g. shifted decimal place, doubling of numbers), and were corrected when found to be in error. Furthermore, we compared data to descriptive entries in annual records of the Hofkeller and to literature [28], [29] to take anthropogenic factors into account. Outliers that could be explained by human decisions on vineyard management (e.g. yield limiting, hand selection) were identified.

After comparison with alternative data sources, the outlier yield of 1812 was identified as a misprint (shifted decimal place in the information on yield) in the literature and was corrected from 48.31 to 4.8 hl/ha. Post World War II records of 1945–7 were excluded from analysis because of wartime damage to the vineyards [28]. The record yield of 1982 was checked against the relevant annual report [30] and proved to be accurate due to a very good growing season. No reasons were found to reject the high must sugar content in 1893. The must sugar content outlier in 1994 appears to have been the consequence of human decisions to focus on high quality throughout the year, implying a strict yield limit, and several rounds of selective harvest by hand [31].

### Climate Data

Owing to its location on south-facing slopes above the River Main, the climate of Lower Franconia is suitable for wine growing and the area has a long history of wine production. However, climate data from the vicinity of Würzburg covering the whole study period do not exist. The local climate stations in Würzburg only date back to 1879 and have been relocated several times. Thus, local climate data neither cover the whole study period nor are homogenous and therefore cannot be used for our analysis.

In a comparison of temperature responses of long-term phenological records, the consequences of choosing either local, national or other European temperatures (i.e. Central England Temperature records compared to German wine data) were considered to be small [19], [32]. Thus, averaged temperature data for the whole of Germany (current boundaries) were used in this study. The homogenised dataset consists of monthly observations of mean temperature (1805–1998) based on Rapp [33], which originally derived from four climate stations in central Europe (Utrecht, Potsdam, Basel and Vienna). Since 1891 homogenised averaged temperature data from German weather stations were used. The number of stations has increased (31 in 1891, 75 in 1951). Since 1997, German SYNOP (surface synoptic observations) have been used. For further information see Rapp [33]. The dataset was extended to include 1999–2010 using monthly observations of homogenised mean temperatures of Germany from the Deutscher Wetterdienst (DWD: German Meteorological Service).

For the period 1962–2010, monthly temperature, precipitation and sunshine on a 1km × 1km grid for each site were obtained from the DWD and the mean of the two grids covering “Stein” and “Leiste” was used for further analysis.

However, this study does not address any possible benefits of increasing atmospheric carbon dioxide concentrations on grapevine yield since those long-term data are not available in sufficient quality.

The oldest continuous record of direct measurements of carbon dioxide started in 1957 [34] and therefore does not cover the whole observation period since 1805. Global CO_2_ models can suffer from questions of robustness and reliability and do not represent CO_2_ levels at local scales [35].

### Statistical Analysis

All calculations were performed with IBM SPSS version 19. To create a continuous homogenised time series of grapevine yield, Juliusspital yields in the overlapping period (1874–1905) were adjusted using linear regression techniques to have an identical mean to that of the Hofkeller in the same period. This adjustment was applied to Juliusspital yields for the years 1906–1914 to extend the Hofkeller time series to include these additional 9 years. In the following, this extended time series will be referred to as Hofkeller period 1 (1805–1914). The three independent time periods (1805–1914, 1915–1952, 1962–2010) were then analysed separately (see Table 1). Yield was converted to must volume and yield per hectare was then calculated using the respective acreage. Since mean values were not provided, the mean must sugar content (1864–1904 and 1962–2010) was approximated from the average of the reported minimum and maximum must sugar content. The two must sugar time series will be referred to as period 1S and period 3S.

All data were tested for compliance with the normal distribution (Kolmogorov-Smirnov test). Relationships between variables were explored using Pearson correlation coefficients. Differences between periods were tested for equality of means using a one-way ANOVA followed by LSD post-hoc tests, and equality of slopes tested using regression methods [36].

Regression techniques were used to relate grapevine variables (yield and must sugar content) to year and climate variables. Quadratic responses to temperature were not a significant improvement over linear responses, so only the latter are considered further. Thus, multiple linear regression procedures, applying a stepwise approach from a null model, were used to select the most significant climate predictors of yield and must sugar content. Potential climate variables were restricted to German mean monthly temperatures. Year was subsequently considered as an additional variable to the final temperature model to look for unexplained trend through time. If this were to result in a significant improvement to the model, it would suggest that factors other than monthly temperature (e.g. management advances) were contributing to changes in yield and quality. We additionally used detrended data to investigate differences in temperature relationships once trends through time were eliminated. To test if the models for the 1962–2010 period could be improved by adding local mean and maximum temperature, sunshine or precipitation, the local climate data were considered as additional variables to the final temperature model based on national temperature data to see if they significantly improved explanatory power.

## Results

### Trends in Yield and Must Sugar Content

**Figure 2:**
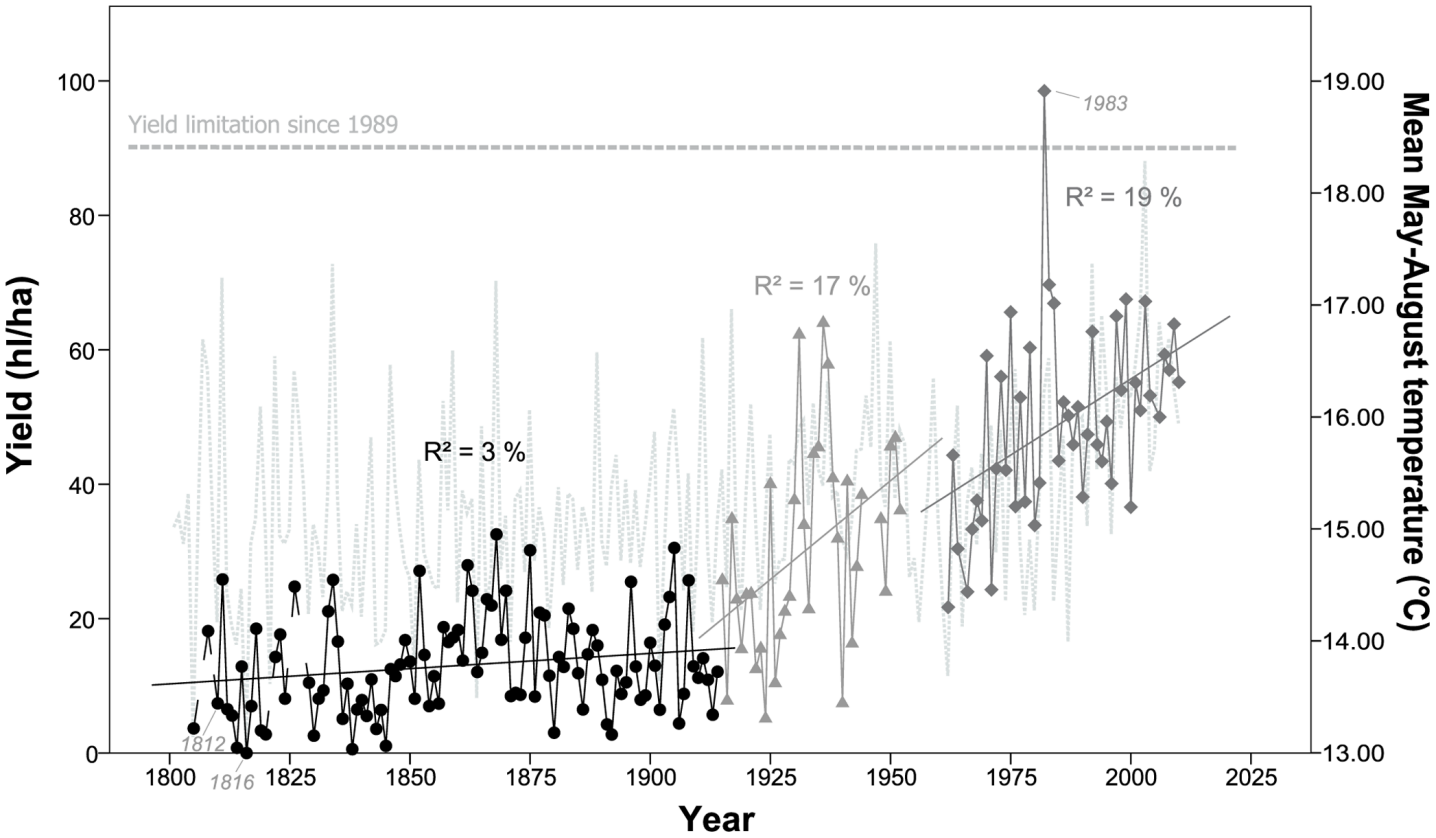
Time series of mean yield (hl/ha), originating from Eifler[24], [54] and Weigand [25] (black solid circles), from Bayerisches Staatsministerum für Ernährung [26] (light grey solid triangles) and annual vintage records (dark grey solid rhombs). Mean April to August temperature are plotted against the right y-axis (dotted line in light grey). Regression lines superimposed. Dotted horizontal line indicates yield limitation (90 hl/ha) in force since 1989.

**Figure 3:**
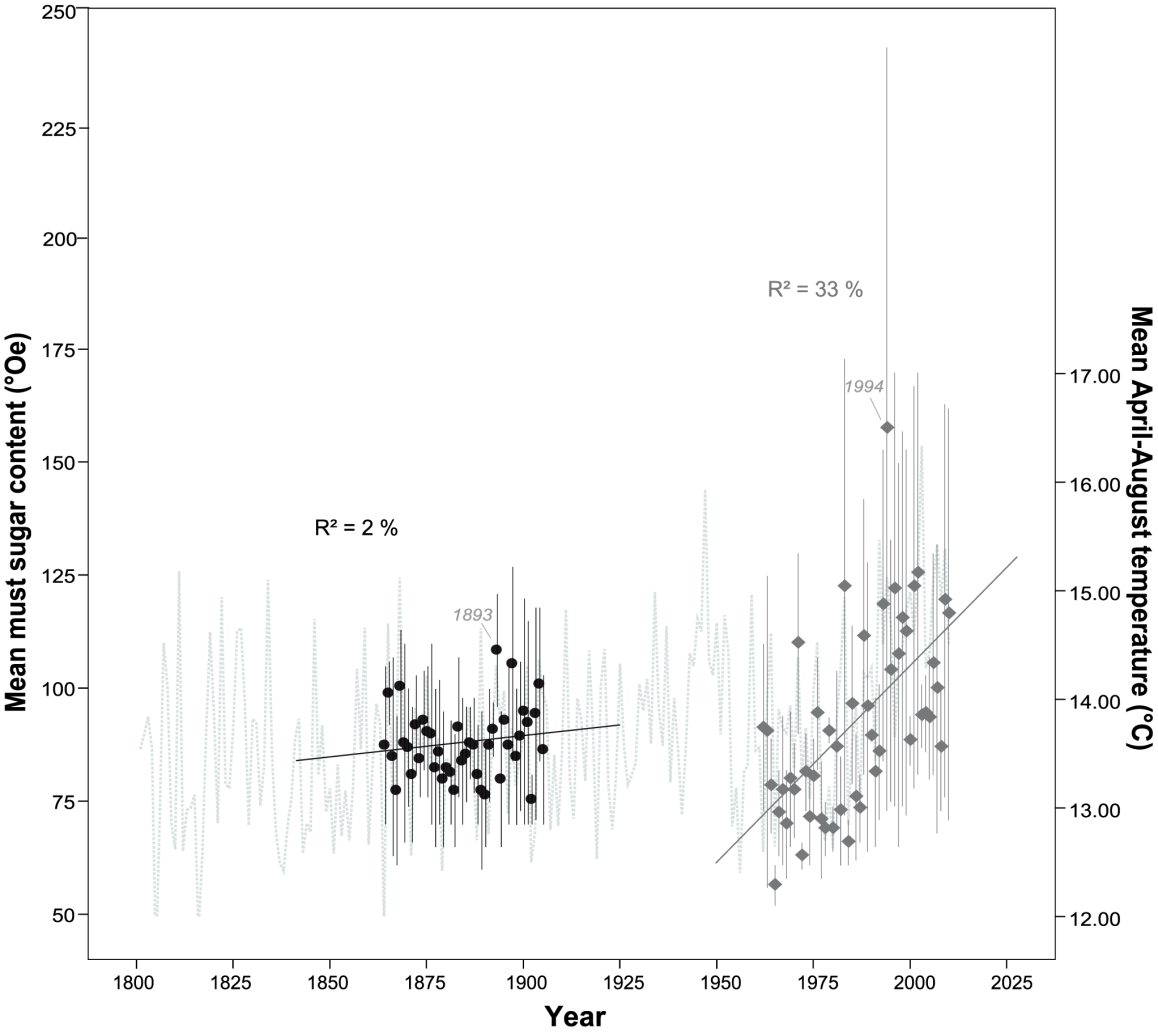
Time series of mean must sugar content (°Oe) (left y-axis), originating from Eifler [24] (black solid circles) and recent annual vintage records (dark grey solid rhombs). Vertical bars connect minimum and maximum must sugar content for each year. Mean April to August temperature are plotted against the right y-axis (dotted line in light grey). Regression lines superimposed.

All time period datasets conformed to a normal distribution (results not shown). The regression between the overlapping datasets (1874–1905) of Juliusspital and Hofkeller resulted in a coefficient of determination (R^2^) of 0.80 (p<0.001). The mean yield of period 1 (1805–1914) was 12.8 hl/ha (±7.5 hl/ha). The increase in yield during this time period was too small to be detected as statistically significant. The yield in period 2 (1915–1952) averaged 30.2 hl/ha (±15.3 hl/ha) and a significant (p<0.05) increase was detected (approximately 6 hl/ha per decade). The average yield in period 3 (1962–2010) was 49.3 hl/ha (±14.3 hl/ha) and increased significantly (p<0.01) by approximately 4.5 hl/ha per decade (Table 2). Significant (p<0.001) differences among mean yields were apparent between all three periods (ANOVA results not shown). Differences in slopes were significant (p<0.001) apart from between periods 2 and 3 (p = 0.599; results not shown). For the whole observation period, yield per hectare averaged 25.4 hl/ha (±19.0 hl/ha) with a highly significant increase (p<0.001) of approximately 2.5 hl/ha per decade equating to approximately 51.25 hl/ha over the last 200 years (Table 2; Figure 2). Must sugar content of period 1S (1864–1905) averaged 87.6°Oe (±7.8°Oe) and did not show a significant trend (Figure 3). The mean must sugar content of period 3S (1962–2010) averaged 92.6°Oe (±20.6°Oe) and displayed a highly significant (p<0.001) increase by 8.3°Oe per decade (Table 2 and Figure 3). While there was no significant difference in means (ANOVA, p = 0.137) between the two periods, the difference in slopes was significant (p<0.001; results not shown). For the whole observation period, must sugar content averaged 90.4°Oe (±16.1°Oe) with a highly significant increase (p<0.001) of approximately 3.3°Oe per decade.

**Table 2:** Summary data for yield (hl/ha) and must sugar content (°Oe).

Yield (hl/ha)	n	Mean	SD	Min	Max	b	R^2^	p
Period 1 (1805–1914)	103	12.8	7.5	0.0	32.5	0.03	0.02	0.160
Period 2 (1915–1952)	35	30.2	15.3	5.1	46.0	**0.59**	**0.17**	**0.012**
Period 3 (1962–2010)	47	49.3	14.3	21.7	98.5	**0.45**	**0.19**	**0.002**
**Mean must sugar content (°Oe)**								
Period 1S (1864–1905)	42	87.6	7.8	75.5	108.5	0.09	0.02	0.377
Period 3S (1962–2010)	49	92.6	20.6	56.5	157.5	**0.83**	**0.33**	**<0.001**
**Overall trend yield (hl/ha)**								
Period 1–3 (1805–2010)	185	25.4	19.0	0.0	98.5	**0.25**	**0.61**	**<0.001**
**Overall trend must sugar content (°Oe)**								
Period 1S & 3S (1864–1905, 1962–2010)	91	90.4	16.1	56.5	157.5	**0.33**	**0.17**	**<0.001**

Data is presented separately for the three periods and for the entire study period. Trends through time are summarized in the final three columns from regressions of the variable on year. b = slope of the regression coefficient. Results in bold are significant (p<0.05).

### Effects of Temperature on Yield and Must Sugar Content

**Figure 4:**
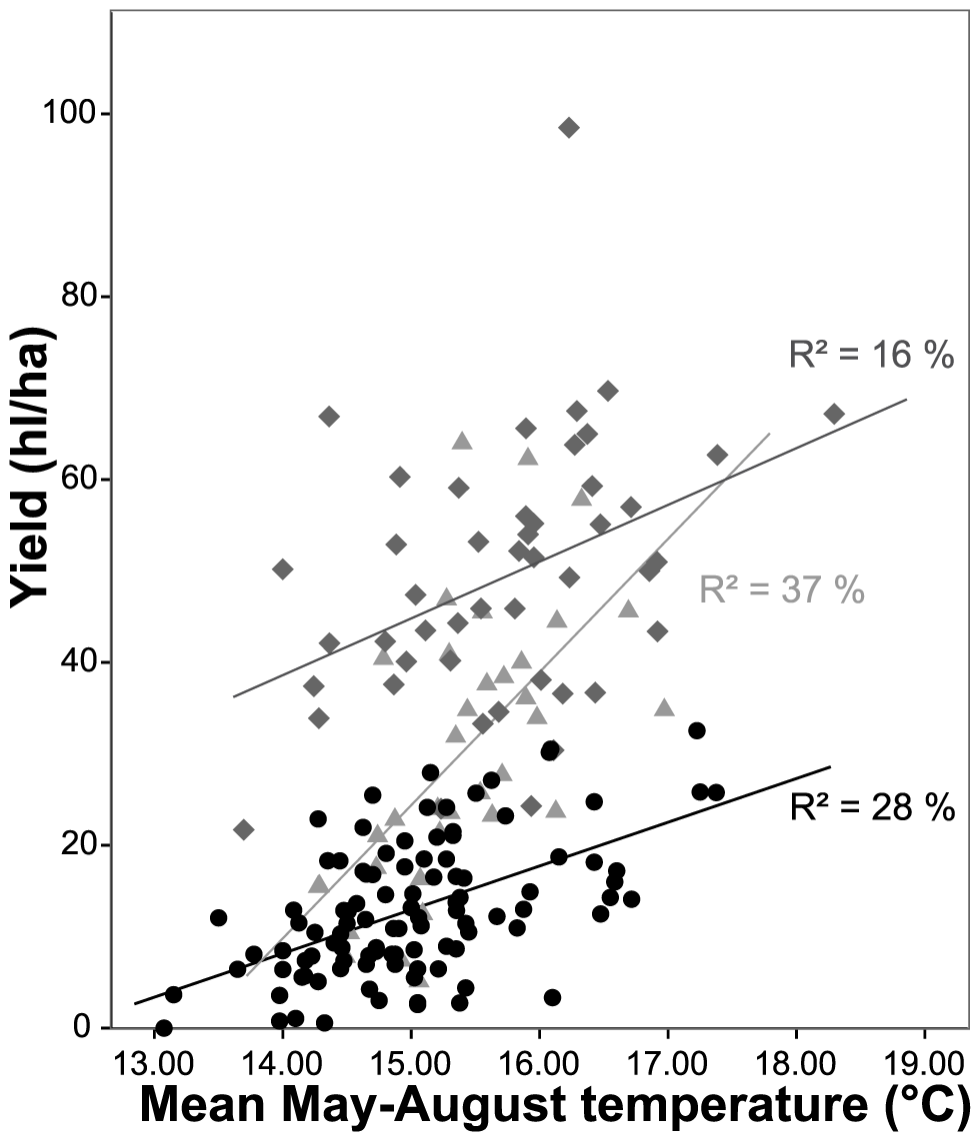
Relationships between yield (hl/ha) and mean May to August temperature (°C). 1805–1914 [24], [25] (black solid circles), 1915–1952 [26] (light grey solid triangles) and 1962–2010 (annual vintage records) (dark grey solid rhombs). Regression lines superimposed.

**Figure 5:**
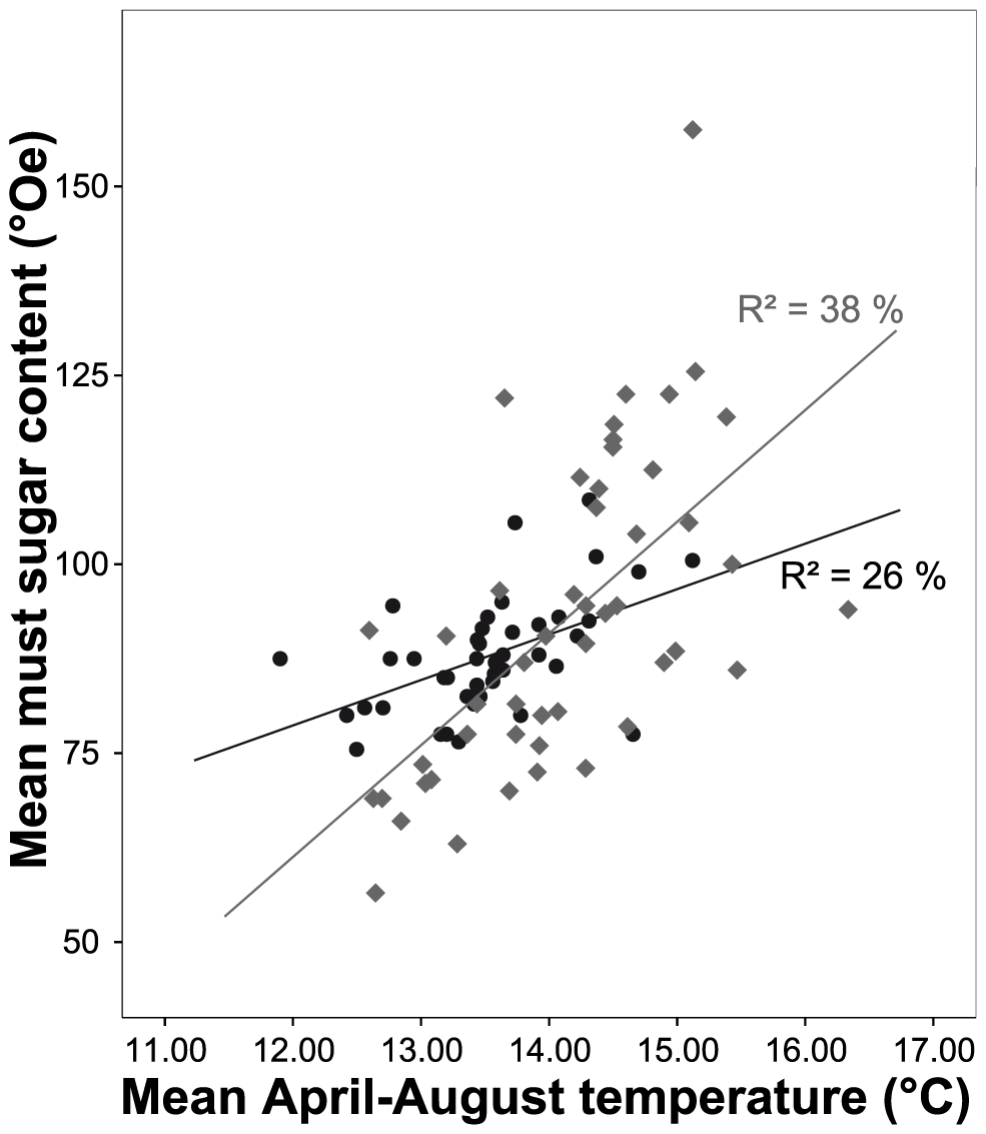
Relationships between must sugar content (°Oe) and mean April to August temperature (°C). 1864–1905 [24] (black solid circles) and 1962–2010 (annual vintage records) (dark grey solid rhombs). Regression lines superimposed.

Mean annual temperatures, averaged across Germany, increased by 1.44°C from 1805 to 2010 (R^2^ = 27%, p<0.001). Multiple regression models revealed significant relationships of yield and must sugar content on German mean temperatures of the preceding months (Figures 4 and 5 show, for simplicity, relationships with mean summer temperatures). Overall, warmer temperatures during the growing season resulted in increased yield and must sugar content. Adding year to the temperature model improved the explanation of the overall trend through time (1805–2010), i.e. trends were present that cannot be explained by changes in mean monthly temperature (Table 3).

**Table 3:** Multiple regression model summaries and regression coefficients of the significant climate variables and when year added (if significant improvement to model).

					DWD German mean temperature (°C)						
	Period	R^2^	Year	Mar	Apr	May	Jun	Jul	Aug	Sep	Oct
**Yield (hl/ha)**	1 (1805–1914)	33% ***				1.08**	2.31***	1.29**			
	2 (1915–1952)	36% **					5.14**		4.82*		
	3 (1962–2010)	28% **							4.48**	3.36*	
	3 (1962–2010)	34%***	0.29 (p = 0.051)						2.95 n.s.	3.17*	
	1–3 (1805–2010)	31% ***		1.14*		1.93*	2.85**	2.63**	3.75***		
	1–3 (1805–2010)	68% ***	0.22***	-0.15 n.s.		0.89 n.s.	2.53***	1.12 n.s.	1.90**		
**Mean must sugar content (°Oe)**	1S (1864–1905)	28% **						2.39**			1.93*
	3S (1962–2010)	43% ***			5.33**			4.54**	4.04*		
	3S (1962–2010)	48%***	0.40 (p = 0.061)		3.62 n.s.			3.83*	2.43 n.s.		
	1S&3S (1864–2010)	35% ***			3.07**			3.65***	3.39***		

Data are presented separately for all periods and overall. Key to significance of coefficients:

* p<0.05,

** p<0.01,

*** p<0.001 and n.s. not significant.

Yield in period 1 was highly significantly related to mean May to July temperature (Table 3, R^2 = ^33%). Of these months, only June and July were modestly correlated with one another (r = 0.225, p = 0.019). An increase of 1°C during these months was associated with an increase of yield of approximately 5 hl/ha. Yields in period 2 were also responsive to temperature, with 36% of the variation in yield being explained. An increase of 1°C during June and August was associated with an increase of yield of approximately 10 hl/ha. Yields in 1962–2010 were significantly related to mean August and September temperature, explaining 28% of the variation. Adding year to this model led to an almost significant (p = 0.051) improvement of the model (R^2^ = 34%), suggesting a non-climate related yield increase. The yield from 1805–2010 was highly responsive (R^2^ = 31%) to temperature. An increase of 1°C during the months March and May to August resulted in an increase of yield of approximately 12 hl/ha. Adding year to this model led to a significant improvement of the model, with 68% of the variation being explained. In this model, June and August temperature retained a very significant impact on yield.

For must sugar content during period 1S, 28% of the variation was explained by the regression model. Temperatures during July and October were significantly related to must sugar content with warmer conditions typically leading to higher levels of must sugar. In the regression model for period 3S, 43% of the variation in must sugar content was explained. Must sugar content during this period appeared to be most influenced by April, July and August temperature. Adding year to this temperature model resulted in a not quite significant improvement (p = 0.061), explaining 47% of the variation. Must sugar content of the whole observation period (1864–2010) was also highly responsive to temperature in April, July and August, with 35% of the variation being explained (Table 3). The use of detrended data of yield and must sugar content only resulted in marked differences in temperature relationships for period 3, modifying the months selected by stepwise regression (results not shown).

### Effects of Local Temperature, Sunshine and Precipitation on Yield and Must Sugar Content

There were highly significant relationships (p<0.001) between German and local temperatures, with correlation coefficients ranging between 0.95 and 0.98. Replacing German mean temperature by local mean or maximum temperature for period 3 and 3S did not result in any improvement to the regression models (Table S1).

Adding local sunshine hours and precipitation to the national mean temperature model for yield in period 3 suggests that increasing sunshine hours in August further increased yield, though at a lower level of significance (p<0.05). This resulted in a significant improvement to the model with 35% of variation being explained. For mean must sugar content, adding June sunshine hours and precipitation also significantly improved the regression model, explaining 61% of the variation. Must sugar content was negatively associated with June precipitation (p<0.001) and at a lower level of significance negatively related to June sunshine (p<0.05). Adding year to the regression model did not reveal any significant improvement to the model (Table 4).

**Table 4:** Multiple regression model summaries and regression coefficients of the significant climate variables for period 3/3S (1962–2010) when local sunshine and precipitation were considered as variables in addition to national temperature models in Table 2.

		German mean temperature (°C)				Local (Würzburg) climate data		
						Sunshine hours		Precipitation sum (mm)
Period 3/3S (1962–2010)	R^2^	Apr	Jul	Aug	Sep	Jun	Aug	Jun
**Yield (hl/ha)**	28% **			4.48**	3.35*			
	35% ***			7.06***	2.94*		0.13*	
**Mean must sugar content (°Oe)**	43% ***	5.33**	4.54**	4.04*				
	61% ***	5.37***	3.98**	4.33*		−0.12*		−0.31***

Key to significance of coefficients:

* p<0.05,

** p<0.01 and ***p<0.001.

## Discussion

Overall, there have been increases in must yield and must sugar content of grapevine over the recording period in the Franconian region of Germany. The trends through time are compatible with other long-term studies [2], [13], [14], [19], [37], [38]. However, as Maurer et al. [9] explained, many factors affect yield and must sugar content. Therefore trends must be interpreted with caution in order to estimate how much of the increase in must yield and must sugar content can be attributed to changes in temperature.

No significant trend in yield was detected during the first period. However, several historical events in this period can be identified. The eruption of Mount Tambora in 1815 caused the following year to be described as a “year without a summer” in Europe [39], [40], resulting in unusual late harvests or crop failures [5], [8], [10], [11], [41], [42]. For the Hofkeller, this resulted in zero yield in 1816. The average yield during 1805–1914 (13.1 hl/ha) was much lower than in wine-growing regions in Switzerland in the early 19^th^ century (26 to 63 hl/ha) [15]. This may be explained by mismanagement in the early years after the Mediatisation (the reorganisation of the German states in the early 19^th^ century), which led to deterioration of the vineyards [43]. A change in cultivars, single cultivar vineyards and later harvesting encouraged steadily increasing yields since the 1840s [43]. Between 1888 and 1902, downy mildew (*Plasmopara viticola*), powdery mildew (*Oidium tuckeri*) and grape phylloxera (*Viteus vitifoliae*) appeared for the first time [43] and contributed to the variability in yield around 1900. In the subsequent period, yield increased by approximately 6 hl/ha per decade. During this period, the former mass production gave way to a strict quality-oriented viticultural policy [28]. Overall increases in yield may be due to improved cultivation methods and changes in grape cultivars, while the large variability can be attributed to the impact of the World Wars. Re-allocation of viticultural land and intensification of production [23] resulted in a significant trend over time (p = 0.002) during period 3 with yield increasing by approximately 4 hl/ha per decade. However, larger grape yields are generally associated with higher economic risk [14] and poorer wine quality by affecting the leaf area/fruit weight ratio [44]. In 1989, in order to produce higher quality and to encourage competitiveness, the German wine law imposed a regulation (Deutsches Weingesetz 1989) on limiting the yield per hectare. For Franconia, the limit for commercial must or wine is 90 hl/ha (Figure 2). Therefore, record yields as in 1983 (98.5 hl/ha) are now prevented by law. However, as confirmed by Jones & Davis (2000) for the Bordeaux region in France, despite regulated controls, production levels are still clearly influenced by trends over time.

The means in yield differed between the three time periods. Apart from periods 2 and 3, differences in slopes were also significant. These differences reflect changes in viticultural practices (i.e. technological advance, the varieties cultivated, or the style of wine produced) [9], [10] and appear to be a logical explanation for trends in yield to be much more obvious in the later periods. In this study, the change over time for period 3 accounted for 19% of the variation in yield. For the same region and approximately the same period (1968–2010), Bock et al. [45] showed that the trend over time accounted for between 11 and 43% of the variation in grape harvest dates, with a trend towards earlier harvesting.

Although there was no significant difference in mean must sugar content, trends through time differed between the two time periods. As in yield, the different trends may reflect the changes in viticultural practices. Period 1S did not have a significant trend while period 3S experienced a highly significant increase in must sugar content. In comparison to Urhausen et al. [37] in the Mosel Valley, the mean must sugar content, its standard deviation and the increase over time was very high (92.6°Oe; ±20.6°Oe; 8.3°Oe increase per decade). This is because the mean must sugar content was calculated as the average of the reported minimum and maximum must sugar content. Special wines (e.g. late harvest or ice wines) with very high must sugar contents strongly influence the mean values. Thus, high must sugar content might not be due to high temperatures during the growing season. Ideally we would have mean must sugar content, but only the minimum and maximum were reported.

The main influential factor for grapevine growth and must sugar accumulation in regions without water limitation is temperature [17], [38]. Yield and must sugar content were significantly influenced by mean temperatures during the growing season. In comparison to Bock et al. [45], where climate variables explained up to 80% of the variation in grape harvest dates for the period 1968–2010, only 28% of the variation in yield for 1962–2010 in the current study could be explained by temperature. While grape harvest dates are dependent on climate over the whole growing season, yield is furthermore dependent on individual weather events (e.g. frost and rain) during flowering, the data for which were not available in the present study. Additionally, Bock et al. [45] predominantly used harvest dates of single cultivars rather than information, such as yield, which combines results from mainly Silvaner and Riesling cultivars. Whilst temperature was the most significant variable in 1805–1914 and 1915–1952, the regression model for 1962–2010 was marginally improved by adding year (p = 0.051), explaining up to 34% of the variation in yield (p<0.001). The difference in mean temperature between 1805–1914 and the last 20 years of time period 3 was 1.3°C and the difference in mean yield was 41 hl/ha. Given the regression equation of period 1 you would expect a 1.3°C increase in temperature to generate a 6.3 hl/ha increase in yield. Therefore a crude estimate of yield increase due to temperature change is approximately 15% of that experienced. This suggests that other factors, such as management and cultivation improvements, and cultivar choice are responsible for the remaining increase in yield and these non-climatic influences are likely to be the most important. In contrast, in an Australian study on wheat yield (1952–1992), 30–50% of the observed increase were estimated to be due to climate trends [46].

Must sugar content is an indicator of ripeness and harvest timing [47] and was significantly related to temperature (Table 3 and Figure 5). The must sugar content of 1864–1905 was related to July and August temperature, explaining 28% of the variation, while the must sugar content of 1962–2010 was highly responsive to April, July and August temperature and explained 43% of the variation. Results of the latter period are confirmed by Bock et al. [45] who found that must sugar content in Franconia was significantly dependent on temperature during pre-flowering (i.e. April) and pre-harvest (i.e. July and August). Adding year to the regression model resulted in a not quite significant (p = 0.061) improvement. The difference in mean temperature (April to August) between 1864–1905 and the last 20 years of period 3 (1991–2010) was 1.3°C. Differences in must sugar content were 19.8°Oe. Given the regression equation for must sugar content in the first time period, a 1.3°C increase in temperature would result in a 7.5°Oe increase of must sugar content. Thus, a crude estimate of sugar increase due to temperature change is approximately 38% of that achieved. Therefore the impact of management and cultivation improvements appear to be slightly less important for the improvements to must sugar content than for yield. The use of detrended data for regression analysis in period 3 modified the months selected by stepwise regression. However, the overall picture of warmer summers leading to higher must sugar and yield remained.

Since local monthly temperature, sunshine and precipitation data for Würzburg were not available for other years, the effect of these climate variables were tested for 1962–2010 only. Menzel et al. [48] found, in a comparison of temperature responses of long-term phenological records, that the consequences of choosing either local or national mean temperatures were considered to be small. This was confirmed in this study, since replacing the national temperature with local temperature did not improve the model for either yield or sugar content. Adding precipitation and sunshine resulted in an improvement to both yield and must sugar content models. For yield, August sunshine had a significant positive effect. For must sugar content, June sunshine and June precipitation had significant negative effects resulting in up to 61% of the variation being explained. The significant negative effect of June precipitation is in line with the findings of other authors [38]. The negative effect of June sunshine was at a lower level of significance and is not confirmed by other studies. However, precipitation and sunshine were frequently less important in grapevine models and typically at a lower level of significance [10], [38], [45].

Free Air CO_2_ Enrichment (FACE) experiments on grapevine, conducted over 2 to 3 growing seasons, have reported positive effects of elevated CO_2_ on grapevine photosynthesis and therefore yield [49], [50]. In contrast, long-term studies of CO_2_ enrichment (FACE) in mature deciduous forest trees have not observed any consistent, significant increases in growth or biomass under elevated CO_2_. Those authors conclude that the initial increase in photosynthesis is down-regulated [51], [52]. Schultz (2000) assumes that long-term exposure to elevated CO_2_ may have similar effects on grapevine [53]. Furthermore, a sudden increase in CO_2_ concentration might lead to a stronger response in trees than a slow and gradual increase of 1 to 3 ppm per year. While positive effects on must sugar levels were reported through the ripening period, at the time of harvest the CO_2_ effect had disappeared [49].

Therefore, carbon dioxide levels were not included in the regression model of this study.

### Conclusions

This study is the first report on long-term data sets of grapevine yield and must sugar content in Germany and confirms an upward trend in yield and must sugar content between 1805 and 2010. The greatest increase in yield was between 1915 and 1952 and is likely due to improved management and cultivation techniques. However, the increase in yield has been limited since the late 1980s, especially due to the introduction of a yield limit in German agricultural policy. Therefore, we distinguish between the impact on yield and composition by anthropogenic factors and temperature. For the recording period, approximately 15% of the increase in yield and 38% of the increase in must sugar content can be attributed to changes in temperature. The relationship between temperature and must sugar content of the third period is much stronger than for yield; probably due to yield limitations put in place by policy changes. Increasing temperatures will require adapted viticultural practices. Due to economic risks, the introduction of the limitation of yield was one step to mitigate further record yields. However, with rising temperature, must sugar content will likely increase in the future. Using local monthly temperature did not significantly improve the models but precipitation and sunshine data did. However, precipitation and sunshine data are not available for the earlier periods. The significant relationships of temperature with yield and must sugar content support the view that these variables, where available historically, could be used as climate proxies or assist in climate reconstructions. However, while grape harvest dates and must sugar content have been confirmed as climate proxies in numerous studies, recent grapevine yields appear to be less reliable due to adapted viticultural techniques. This study suggests that yield data may be used cautiously for calibrating yield-temperature functions for the pre-instrumental period.

## Supporting Information

Table S1 **Multiple regression model summaries and regression coefficients of the significant temperature variables for period 3/3S (1962–2010), using either national mean temperature, local mean temperature or local maximum temperature.** Key to significance of coefficients: *p<0.05, **p<0.01 and ***p<0.001.
(DOC)

## References

[1] van Leeuwen C, Friant P, Chone X, Tregoat O, Koundouras S, et al (2004) Influence of climate, soil, and cultivar on terroir. Am J Enol Vitic 55: 207–217.

[2] Jones GV, White MA, Cooper OR, Storchmann K (2005) Climate change and global wine quality. Clim Change 73: 319–343. DOI 10.1007/s10584–005–4704–2

[3] Seguin B, de Cortazar IG (2005) Climate warming: Consequences for viticulture and the notion of ‘terroirs’ in Europe. Acta Hort 689: 61–69.

[4] Webb LB, Whetton PH, Barlow EWR (2008) Climate change and winegrape quality in Australia. Clim Res 36: 99–111.

[5] Chuine I, Yiou P, Viovy N, Seguin B, Daux V, et al (2004) Historical phenology: Grape ripening as a past climate indicator. Nature 432: 289–290. 15549085 10.1038/432289a

[6] Menzel A (2005) A 500 year pheno-climatological view on the 2003 heatwave in Europe assessed by grape harvest dates. Meteorol Z 14: 75–77.

[7] Maurer C, Koch E, Hammerl C, Hammerl T, Pokorny E (2009) BACCHUS temperature reconstruction for the period 16th to 18th centuries from Viennese and Klosterneuburg grape harvest dates. J Geophys Res-Atmos 114.

[8] Kiss A, Wilson R, Bariska I (2010) An experimental 392-year documentary-based multi-proxy (vine and grain) reconstruction of May-July temperatures for Kõszeg, West-Hungary. Int J Biometeorol 55: 595–611. 20953886 10.1007/s00484-010-0367-4

[9] Maurer C, Hammerl C, Koch E, Hammerl T, Pokorny E (2011) Extreme grape harvest data of Austria, Switzerland and France from A.D. 1523 to 2007 compared to corresponding instrumental/reconstructed temperature data and various documentary sources. Theor Appl Climatol 106: 55–68.

[10] Garnier E, Daux V, Yiou P, de Cortazar-Atauri IG (2011) Grapevine harvest dates in Besancon (France) between 1525 and 1847: Social outcomes or climatic evidence? Clim Change 104: 703–727.

[11] Etien N, Daux V, Masson-Delmotte V, Mestre O, Stievenard M, et al (2009) Summer maximum temperature in northern France over the past century: instrumental data versus multiple proxies (tree-ring isotopes, grape harvest dates and forest fires). Clim Change 94: 429–456.

[12] Webb LB, Whetton PH, Bhend J, Darbyshire R, Briggs PR, et al (2012) Earlier wine-grape ripening driven by climatic warming and drying and management practices. Nature Clim Change 2: 259–264.

[13] Santos J, Malheiro A, Karremann M, Pinto J (2011) Statistical modelling of grapevine yield in the Port Wine region under present and future climate conditions. Int J Biometeorol 55: 119–131. 20461417 10.1007/s00484-010-0318-0

[14] Bindi M, Fibbi L, Gozzini B, Orlandini S, Miglietta F (1996) Modelling the impact of future climate scenarios on yield and yield variability of grapevine. Clim Res 7: 213–224.

[15] Pfister C (1981) Die Fluktuationen der Weinmosterträge im schweizerischen Weinland vom 16. bis ins frühe 19. Jahrhundert : klimatische Ursachen und sozioökonomische Bedeutung. Schweiz Z Gesch 31: 445–491.

[16] Lauer W, Frankenberg P (1986) Zur Rekonstruktion des Klimas im Bereich der Rheinpfalz seit Mitte des 16. Jahrhunderts mit Hilfe von Zeitreihen der Weinquantität und Weinqualität. In: Frenzel B, editors. Paläoklimaforschung. Mainz: Akademie der Wissenschaften und der Literatur.

[17] Duchêne E, Schneider C (2005) Grapevine and climatic changes: a glance at the situation in Alsace. Agron Sustain Dev 25: 93–99.

[18] Laget F, Tondut JL, Deloire A, Kelly MT (2008) Climate trends in a specific Mediterranean viticultural area between 1950 and 2006. J Int Sci Vigne Vin 42: 113–123.

[19] Storchmann K (2005) English weather and Rhine wine quality: An ordered probit model. J Wine Res 16: 105–120.

[20] Pfister C (1992) Monthly temperature and precipitations in central Europe 1525–1979: quantifying documentary evidence on weather and its effects. In: Raymond S.Bradley, Philip D.Jones, editors. Climate Since A.D. 1500. London: Routledge. 118–152.

[21] Meier N, Rutishauser T, Pfister C, Wanner H, Luterbacher J (2007) Grape harvest dates as a proxy for Swiss April to August temperature reconstructions back to AD 1480. Geophys Res Lett 34.

[22] de Cortazar-Atauri IG, Daux V, Garnier E, Yiou P, Viovy N, et al (2010) Climate reconstructions from grape harvest dates: Methodology and uncertainties. Holocene 20: 599–608.

[23] Robinson J (2006) The Oxford Companion to Wine. Oxford: Oxford University Press. 813 p.

[24] Eifler E (1908) Das ärarialische Weingut in Unterfranken 1805–1905. Leipzig: Deichert. 153. p.

[25] Weigand E (1925) 50 Jahre Weingut & Weinbau im Julius-Hospital zu Würzburg: 1874–1924. Würzburg: 191 p.

[26] Bayerisches Staatsministerium für Ernährung LuF (1977) Festschrift Zum 75jährigen Bestehen Der Bayerischen Landesanstalt Für Weinbau Und Gartenbau. -220.

[27] Klopsch W ed. (2002) 100 Jahre LWG: Bayerische Landesanstalt für Weinbau und Gartenbau Würzburg, Veitshöchheim. Würzburg: Bayerische Landesanstalt für Weinbau und Gartenbau.

[28] Weisensee B (1982) Winzers Freud - Winzers Leid. Der fränkische Weinbau und seine Ernten in 1200 Weinjahren. Witterung - Menge - Güte. Würzburg: Echter. 104 p.

[29] Höllerl H, Schmitt A (1997) Das neue Buch vom Frankenwein. Würzburg: Echter Verlag Würzburg. 216 p.

[30] Bayerische Landesanstalt für Weinbau und Gartenbau (1982) Jahresbericht 1981/82.

[31] Bayerische Landesanstalt für Weinbau und Gartenbau (1994) Jahresbericht 1994.

[32] Menzel A, Estrella N, Testka A (2005) Temperature response rates from long-term phenological records. Clim Res 30: 21–28.

[33] Rapp J (2000) Konzeption, Problematik und Ergebnisse klimatologischer Trendanalysen für Europa und Deutschland. Ber Dtsch Wetterd 212.

[34] Keeling CD, Bacastow RB, Bainbridge AE, Ekdahl CA, Guenther PR, et al (1976) Atmospheric Carbon-Dioxide Variations at Mauna-Loa Observatory, Hawaii. Tellus 28: 538–551.

[35] Chapman SC, Stainforth DA, Watkins NW (2013) On estimating local long-term climate trends. Philosophical Transactions of the Royal Society A-Mathematical Physical and Engineering Sciences 371. 10.1098/rsta.2012.0287 PMC3638378 23588048

[36] Draper NR, Smith H (1998) Applied Regression Analysis. New York: Wiley.

[37] Urhausen S, Brienen S, Kapala A, Simmer C (2011) Climatic conditions and their impact on viticulture in the Upper Moselle region. Clim Change 109: 349–373.

[38] Jones GV, Davis RE (2000) Climate influences on grapevine phenology, grape composition, and wine production and quality for Bordeaux, France. Am J Enol Vitic 51: 249–261.

[39] Oppenheimer C (2003) Climatic, environmental and human consequences of the largest known historic eruption: Tambora volcano (Indonesia) 1815. Prog Phys Geog 27: 230–259.

[40] Fischer EM, Luterbacher J, Zorita E, Tett SFB, Casty C, et al.. (2007) European climate response to tropical volcanic eruptions over the last half millennium. Geophys Res Lett 34.

[41] Shabalova MV, van Engelen AGV (2003) Evaluation of a reconstruction of winter and summer temperatures in the low countries, AD 764–1998. Clim Change 58: 219–242.

[42] Luterbacher J, Dietrich D, Xoplaki E, Grosjean M, Wanner H (2004) European seasonal and annual temperature variability, trends, and extremes since 1500. Science 303: 1499–1503. 15001774 10.1126/science.1093877

[43] Schenk W (1992) Viticulture in Franconia along the River Main: human and natural influences since AD 700. J Wine Res 3 (3): 105–121.

[44] Jackson DI, Lombard PB (1993) Environmental and Management-Practices Affecting Grape Composition and Wine Quality - A Review. Am J Enol Vitic 44: 409–430.

[45] Bock A, Sparks T, Estrella N, Menzel A (2011) Changes in the phenology and composition of wine from Franconia, Germany. Clim Res 50: 69–81.

[46] Nicholls N (1997) Increased Australian wheat yield due to recent climate trends. Nature 387: 484–485.

[47] Conde C, Silva P, Fontes N, Dias ACP, Tavares RMSMJ, et al (2007) Biochemical changes throughout grape berry development and fruit and wine quality. Food 1: 1–22.

[48] Menzel A, Von Vopelius J, Estrella N, Schleip C, Dose V (2006) Farmers’ annual activities are not tracking the speed of climate change. Clim Res 32: 201–207.

[49] Bindi M, Fibbi L, Miglietta F (2001) Free Air CO2 Enrichment (FACE) of grapevine (Vitis vinifera L.): II. Growth and quality of grape and wine in response to elevated CO2 concentrations. European Journal of Agronomy 14: 145–155.

[50] Moutinho-Pereira J, Goncalves B, Bacelar E, Cunha JB, Coutinho J, et al (2009) Effects of elevated CO2 on grapevine (Vitis vinifera L.): Physiological and yield attributes. Vitis 48: 159–165.

[51] Korner C, Asshoff R, Bignucolo O, Hattenschwiler S, Keel SG, et al (2005) Carbon flux and growth in mature deciduous forest trees exposed to elevated CO2. Science 309: 1360–1362. 16123297 10.1126/science.1113977

[52] Bader MKF, Siegwolf R, Korner C (2010) Sustained enhancement of photosynthesis in mature deciduous forest trees after 8 years of free air CO2 enrichment. Planta 232: 1115–1125. 20700744 10.1007/s00425-010-1240-8

[53] Schultz H (2000) Climate change and viticulture: A European perspective on climatology, carbon dioxide and UV-B effects. Aust J Grape Wine Res 6: 2–12 10.1111/j.1755–0238.2000.tb00156.x.

[54] Asshoff R, Zotz G, Korner C (2006) Growth and phenology of mature temperate forest trees in elevated CO2. Glob Change Biol 12: 848–861.

