# Supplementary material for: Climate-Induced Changes in Grapevine Yield and Must Sugar Content in Franconia (Germany) between 1805 and 2010
Source: PLoS One. 2013 Jul 23;8(7):e69015. doi: 10.1371/journal.pone.0069015 (PMC3720875; doi:10.1371/journal.pone.0069015)
Supplement: Table S1 — Multiple regression model summaries and regression coefficients of the significant temperature variables for period 3/3S (1962–2010), using either national mean temperature, local mean temperature or local maximum temperature. Key to significance of coefficients: *p<0.05, **p<0.01 and ***p<0.001. (DOC) [file pone.0069015.s001.doc]

**Supporting information table**

Table S1. Multiple regression model summaries and regression coefficients of the significant temperature variables for period 3/3S (1962-2010), using either national mean temperature, local mean temperature or local maximum temperature. Key to significance of coefficients: *p<0.05, **p<0.01 and ***p<0.001.

| **Period 3/3S (1962-2010)** | | | | | | | |
| --- | --- | --- | --- | --- | --- | --- | --- |
|  | **Yield (hl/ha)** | | | **Mean must sugar content (°Oe)** | | | |
|  | **R²** | **Aug** | **Sep** | **R²** | **Apr** | **Jul** | **Aug** |
| **National (German) mean temperature (°C)** | 28%** | 4.48** | 3.35* | 43%*** | 5.33** | 4.54** | 4.04* |
| **Local (Würzburg) mean temperature (°C)** | 22%** | 3.37* | 3.14* | 38%*** | 4.46** | 4.14** | 4.00* |
| **Local (Würzburg) max temperature (°C)** | 15%** |  | 2.89** | 36%*** | 2.79* | 3.18** | 3.15* |
